# Concurrent climate extremes in the key wheat producing regions of the world

**DOI:** 10.1038/s41598-019-41932-5

**Published:** 2019-04-02

**Authors:** Andrea Toreti, Ottmar Cronie, Matteo Zampieri

**Affiliations:** 1 European Commission, Joint Research Centre (JRC), Ispra, Italy; 2 Department of Mathematics & Mathematical Statistics, Umeå University, Umeå, Sweden

## Abstract

Climate extremes have profound impacts on key socio-economic sectors such as agriculture. In a changing climate context, characterised by an intensification of these extremes and where the population is expected to grow, exposure and vulnerability must be accurately assessed. However, most risk assessments analyse extremes independently, thus potentially being overconfident in the resilience of the socio-economic sectors. Here, we propose a novel approach to defining and characterising concurrent climate extremes (i.e. extremes occurring within a specific temporal lag), which is able to identify spatio-temporal dependences without making any strict assumptions. The method is applied to large-scale heat stress and drought events in the key wheat producing regions of the world, as these extremes can cause serious yield losses and thus trigger market shocks. Wheat regions likely to have concurrent extremes (heat stress and drought events) are identified, as well as regions independent of each other or inhibiting each other in terms of these extreme events. This tool may be integrated in all risk assessments but could also be used to explore global climate teleconnections.

## Introduction

**Figure 1:**
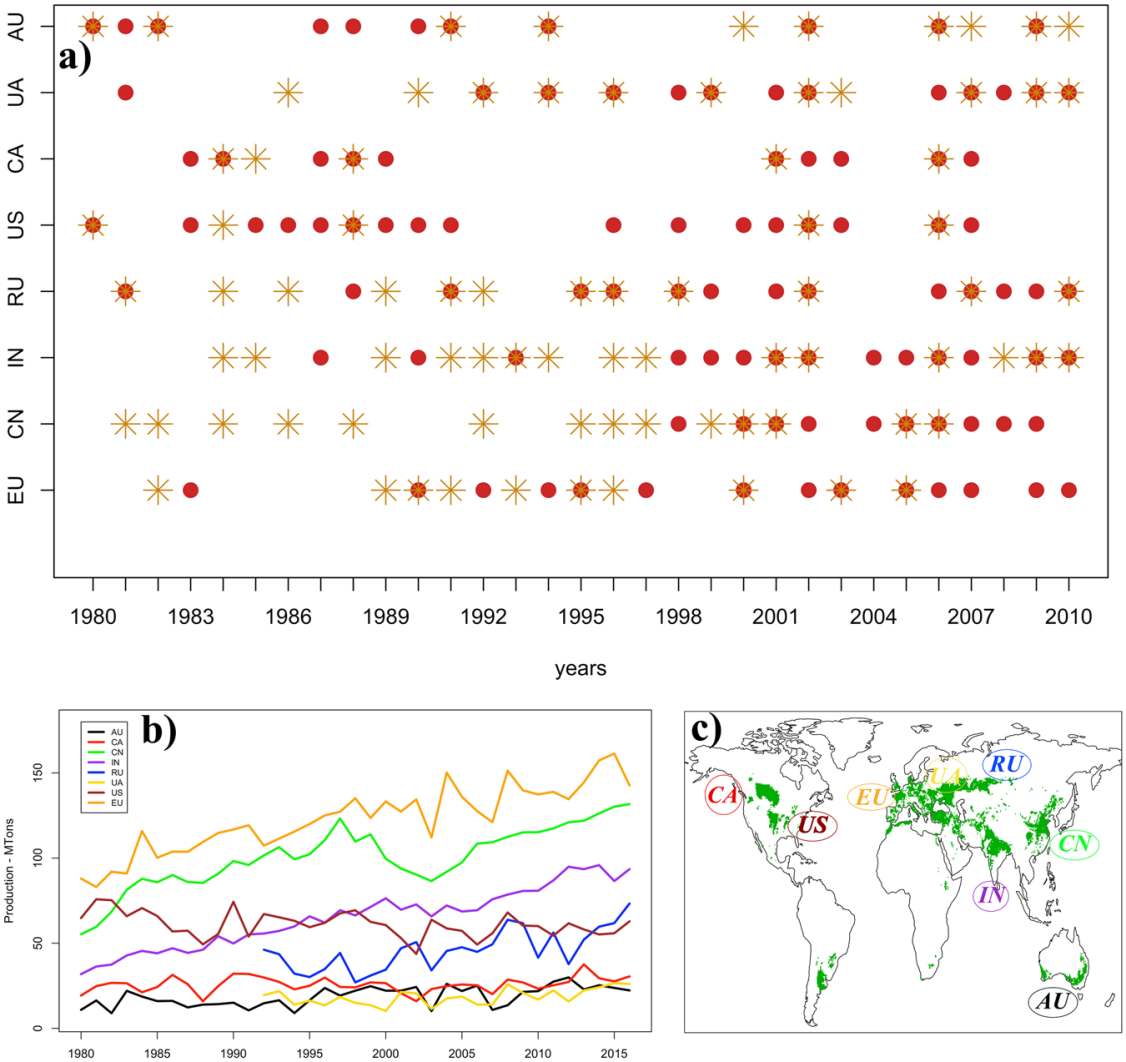
Panel (a): Identified large-scale heat stress events (red circles) and drought (yellow stars) from 1980 to 2010. Panel (b): Wheat production of the 8 key regions of the world from 1980 to 2016 (data from FAOSTAT). Panel (c): Spatial overview of the main wheat producing areas of the world (Data from MIRCA2000).

Climate extremes have an impact in all sectors of socio-economic systems, causing large losses, damages and fatalities. As a consequence, in the last 5 years (2013–2017) more than 672 billion US Dollars of damages have been reported with almost 1 billion people having been affected^1^. Human and natural systems are often able to absorb a single event but they might be exposed and vulnerable to concurrent extremes. For instance, global agricultural markets could be resilient against single country yield failure, but a shock induced by more failures could be difficult to counterbalance and could create negative effects acting on longer time scales. This is the case of global wheat production. Wheat is one of the world’s most important crops and its production in 2016 reached 750 Mtonnes with 220 Mha of harvested area (FAOSTAT). However, most of the production comes from 8 key regions of the world, which accounted for 78% of the global production in 2016 (Fig. 1 and Table 1 in the Supplementary material). Wheat is exposed and vulnerable to extremes occurring in critical phenological phases^2–6^. Events such as drought, heat stress and water excess have all been shown to have detrimental effects on wheat^3,7,8^. Heat stress affects wheat development, photosynthesis, and pollen fertility^8^. Drought severely affects plant growth and processes such as photosynthesis, assimilation, leaf development and enzymatic activity during the grain filling period^7^. These extremes can cause large yield losses and thus have serious socio-economic impacts, especially when occurring in more producing regions within a relatively short temporal interval. It has been shown that drought and extreme heat reduced cereal production at national scale by 9–10%^9^. Furthermore, by looking at long-term behaviour of global wheat yield data and regional time series, heat and water stresses have been shown to explain more than 40% of the yield inter-annual variability^3^. As climate extremes could induce shocks in the global food market, the need of more research (aiming at a better characterisation of these events) has been recently pointed out^10^.

Extreme events, such as heat waves and drought, have been extensively investigated and characterised by using a range of different approaches, most of them classifiable into two broad categories: index-based^11,12^ and Extreme-Value-Theory-based (EVT-based^13^). However, most of the studies analysing these events at the global scale have focused on a single type of event, assuming spatio-temporal independence and in some cases a complete random (Poisson process) behaviour. These two assumptions are quite strict ones and can affect subsequent risk and impact assessments. Dependence is known to be difficult to deal with, especially in the case of extremes. Within the EVT framework, different approaches have been developed, which have mainly been suited for low-dimensional problems^14^, while copula-based approaches have also been applied to characterise compound events^15^. Often, the term ‘compound’ has been used to identify the concomitant (within an a priori chosen temporal lag) occurrence of events (extremes or not) leading to harmful events (considered as extreme) of socio-economic relevance. The summer of 2010 in Russia may be taken as an example of such an event, where dry conditions, fires, pollution and anomalous heat contributed to the extreme event in question. However, a broader definition of compound climate events has recently been proposed^16^. Here, we focus on a class of extremes that could be seen as a subset of compound events, i.e. concurrent extremes. These events are represented by extremes of different types occurring within a specific temporal lag, either in different locations or at the same one, as well as by extremes of the same type occurring in two different locations within a specific time period. Concurrent climate extremes pose a serious threat in terms of potential impacts in key socio-economic sectors which are highly interconnected at the global scale.

To achieve a better understanding of concurrent extremes, while avoiding any a priori strict assumptions of (in)dependence, complete randomness behaviour and homogeneity of the risk of an event, we propose an innovative non-parametric statistical approach. Then, we use this approach to investigate two types of extremes (namely large-scale heat stress and drought) that have occurred in the 8 key wheat producing regions of the world between 1980 and 2010 (Fig. 1 and Table 1 in the Supplementary material). We analyse the dependence of both heat stress and drought events within each of the eight regions, the dependence among different regions and finally their inter-dependence. Thus, the outcomes of this investigation provides insight into the probability of experiencing: heat stress/drought events when one has already occurred within a region, heat stress/drought events in a region when one has already occurred in another one.

Despite the specific case study shown here, we stress that this approach can be directly applied to a vast range of problems where concurrent extremes play an important role.

## The proposed approach

Consider *n*_*R*_ ≥ 2 spatially separated reference regions with labels in ${\mathscr{W}}=\{1,\ldots ,{n}_{R}\}$ (e.g. the key wheat producing regions) and for each, consider a collection of *n*_*C*_ ≥ 1 spatio-temporal measurement functions $\{{Z}_{c,r}(x,t):x\in { {\mathcal R} }^{2}\cap {W}_{r},t\in  {\mathcal R} \}$, $c\in {\mathscr{C}}=\{1,\ldots ,{n}_{C}\}$, $r\in {\mathscr{W}}$, with ${\mathcal R}$ denoting the real line and *W*_*r*_ denoting the spatial region with label *r*. These functions describe spatio-temporal evolutions of some measured quantities, e.g. heat accumulation and precipitation anomalies. In many applications, these quantities are likely to be dependent. Given a threshold *z*_*c*_ for each label $c\in {\mathscr{C}}$ (i.e. setting a threshold to identify local extreme events), we can then study exceedances over time in a specific region *W*_*r*_, and quantify the corresponding temporally evolving spatial extension, by considering some suitable measure *m* of the time-dependent set ${E}_{c,r}(t)=\{x\in {W}_{r}|\,{Z}_{c,r}(x,t)\ge {z}_{c}\}\subseteq {W}_{r}$. Now, by setting a threshold *s*_*c*_ for this measure, we can focus on large-scale extremes, namely the ones satisfying *m*(*E*_*c*,*r*_(*t*)) ≥ *s*_*c*_. Each time point *t* when this occurs we treat as the occurrence of a large-scale event, and the totality of all such events constitutes the collection ${\{{t}_{i}\}}_{i=1}^{n}$ which, together with their associated region and type labels, may be summarised as ${\{({t}_{i},{r}_{i},{c}_{i})\}}_{i=1}^{n}\subseteq  {\mathcal R} \times {\mathscr{W}}\times {\mathscr{C}}$, where *n* ≥ 0 is the total number of exceedances observed. This constitutes a so-called marked (multi-type) temporal point pattern; the mark (*r*_*i*_, *c*_*i*_) contains information about the region of occurrence and the type of event associated with the *i*th occurrence.

Point patterns are modelled by point processes^17,18^ which, roughly speaking, are generalised random samples for which the points may be dependent and the total point count may be random. In particular, the data structure above may be modelled by a multi-type point process

$$Y={\{({t}_{i},{r}_{i},{c}_{i})\}}_{i=1}^{N}\subseteq \, {\mathcal R} \times {\mathscr{W}}\times {\mathscr{C}}.$$

In the framework of so-called intensity-reweighted moment stationarity (see section Methods), we use the so-called marked inhomogenous *J*-function summary statistic^19^ to characterise and identify concurrent extremes. Given non-empty subsets ${B}_{1},{B}_{2}\subseteq {\mathscr{W}}$ and ${C}_{1},{C}_{2}\subseteq C$, the marked (*B*_1_, *C*_1_) to (*B*_2_, *C*_2_) *J*-function is given by

$${J}_{({B}_{1},{C}_{1})\to ({B}_{2},{C}_{2})}(d)=\frac{1-{G}_{({B}_{1},{C}_{1})\to ({B}_{2},{C}_{2})}(d)}{1-{F}_{({B}_{2},{C}_{2})}(d)},\,d\ge 0.$$

Here *F* is the so-called marked inhomogeneous empty space function, which essentially provides the probability of finding a *Y*-point with marks in (*B*_2_, *C*_2_) in a *d*-radius neighbourhood of an arbitrary position $t\in  {\mathcal R}$ on the temporal line (having compensated for the temporally varying event rate). In contrast, the marked inhomogeneous nearest neighbour distance distribution function *G* essentially describes the probability of finding a *Y*-point with mark in (*B*_2_, *C*_2_) in a *d*-radius neighbourhood of a *Y*-point with arbitrary position *t* and mark (*B*_1_, *C*_1_) (having compensated for the temporally varying event rate). Hence, the ratio *J* returns the increase/decrease in probability generated by conditioning on there being a (*B*_1_, *C*_1_)-marked point at *t*. As for Poisson processes, which represent the scenario of no interaction, the *J*-function returns 1 for all ranges *d* > 0; when the *J*-function is smaller than 1 we speak of clustering/aggregation and when it is larger than 1 we speak of inhibition/regularity. As ${J}_{({B}_{1},{C}_{1})\to ({B}_{2},{C}_{2})}(\cdot )$ is non-symmetric in (*B*_1_, *C*_1_) and (*B*_2_, *C*_2_), in practice it is advised to also reverse the order of the two when analysing inherent interactions. To exemplify, note that by setting *B*_1_ = {*i*}, *B*_2_ = {*j*}, $i,j\in {\mathscr{W}}$, *i* ≠ *j*, and *C*_1_ = {1} (e.g. heat stress event), *C*_2_ = {2} (e.g. drought event), we would analyse temporal interactions between heat stress occurrences in key region *i* and drought occurrences in key region *j*. In addition, setting *B*_1_ = {*i*} = *B*_2_ we may study interactions between events that occur in region *i*.

Observed events are often censored in the sense that they are recorded on a discrete time scale and/or the time of occurrence is not completely known or cannot be completely attributed; e.g., only the year of occurrence is known or can be attributed. In other words, the exact times *t*_*i*_ are unknown. To deal with this censoring, we propose to (1) randomly perturb the observed occurrence times, and (2) estimate the above-described summary statistics based on the perturbed data. By repeating this procedure a large number of times (here 100) we obtain an ensemble of estimated *J*-functions, from which we may take the *d*-point-wise median to obtain a final estimate. Note that by re-estimating the *J* summary statistics based on the perturbed patterns and considering their medians, we quantify the structural information in the data. Clearly, the more randomisation/perturbations the better since the underlying idea is that the procedure converges. We here choose to use 100 randomisations as we have empirically observed a stabilisation of the ensemble for already much fewer randomisations.

**Figure 2:**
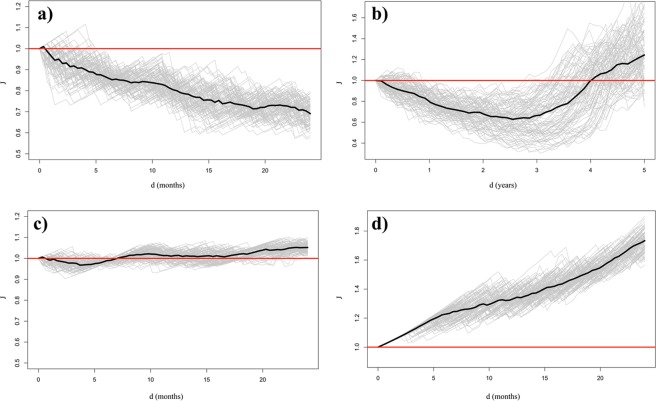
How to use and interpret the J-function. Panels (a) and (b): Analysis of the large-scale heat stress events that have occurred in the wheat areas of China and India (conditioning on China events). Panel (a): Estimated J-functions based on perturbed occurrence times at the monthly scale. Panel (b): Estimated J-functions based on perturbed occurrence times at the annual scale. Panel (c): Estimated J-functions for drought events in the wheat producing regions of India and heat stress events in the wheat producing region of Ukraine (conditioning on Indian events). Panel (d): Estimated J-function for heat stress events in the wheat producing regions of India. Bold lines represent the median of the ensemble. In all cases the perturbation is uniformly distributed.

To clarify how the proposed approach works in practice, Fig. 2 shows how the estimated *J*-function can be used to investigate large-scale extremes (heat stress and drought) in wheat producing regions of the world. Heat stress during the grain filling period is defined as in Zampieri *et al*.^3^, while drought is defined by using the Standardised Precipitation Evapotranspiration Index^20^ at 6-month time scale (see section Methods). As for large scale heat stress events in the wheat areas of China and India, the estimated *J*-function is clearly indicating clustering: having compensated for the temporally varying event rate, the estimated probability of observing a large-scale heat stress event in one of the two countries is higher when the same type of event has already occurred in the other one. The annual and monthly (perturbation range) *J*-function estimates show the temporal time scale of this dependence: 0–3 years (Fig. 2). Concerning large scale drought events in India and heat stress events in Ukraine, the estimates of the *J*-function show Poisson process behaviour, thus these events are interpreted as not influencing each other (Fig. 2). While the estimated *J*-function associated with heat stress events in the wheat areas of India clearly points to inhibition, i.e., taking the varying event rate into account, there is a lower probability of observing a large-scale heat stress event once one has already occurred in the region (Fig. 2). Other examples of inhibition and lack of interaction are shown, respectively, in Figs S1 and S2 of the Supplementary material.

## Wheat producing regions and extremes

Here, we focus on the 8 key wheat producing regions of the world (Fig. 1) and on the two main types of events having proved to cause heavy wheat losses^3,9^, namely heat stress and drought (see section Methods). As described in the previous section, the proposed approach is applied to identify whether the occurrence of large-scale extreme events in one region is influenced by extremes occurring in another region (spatio-temporal dependence) or within the region itself (temporal dependence). Having compensated for the non-stationarity in the intensity functions, three outcomes are therefore possible: no influence, i.e. Poisson process behaviour and thus no dependence; dependence with clustering (or aggregation), i.e. there is higher probability of observing an event if one has already occurred; dependence with inhibition, i.e. there is lower probability of observing an event if one has already occurred.

**Figure 3:**
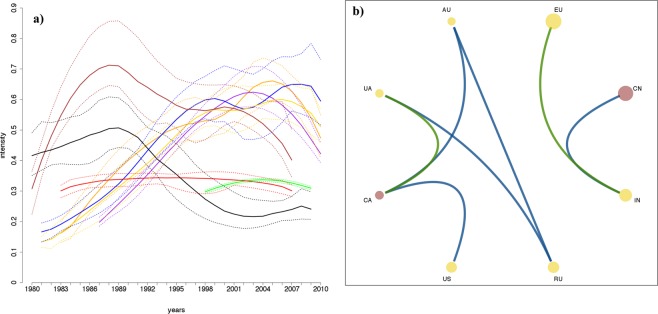
Panel (a): estimated intensity functions of the key wheat producing regions. Bold lines represent the median of the ensemble, while the dotted lines identify the maximum and the minimum of the ensemble. The different colours are associated with the 8 key regions, as in Fig. 1. Panel (b): summary plot of the analysis on heat stress events. Disks represent the 8 key regions, with the size being proportional to the corresponding wheat production in 2016 (data from FAOSTAT). Yellow disks represent regions with temporal inhibition within the region itself, while red ones represent regions with temporal clustering within the region itself. Blue links represent estimated clustering between regions, while green links represent estimated inhibition between regions.

By looking at all the regions of interest and focusing on heat stress events, clear dependence structures emerge (Fig. 3). Two groups can be easily identified: one composed of the three main producers (the European Union, EU, China and India), and another one composed of the remaining countries. As for the former (Fig. 3), China and India have aggregating heat stress events while events in the EU and India seem to inhibit each other. Expected links of dependence are also shown in Fig. 3, e.g. between the U.S. and Canada, Ukraine and Russia. While other ones, such as Australia-Russia and Australia-Canada deserve in-depth dedicated analysis of the large-scale dynamics that could trigger such effects. In almost all regions (except for China and Canada) heat stress events show temporal inhibition. The importance of not having made any homogeneity assumption about the event rate is also evident in Fig. 3, which shows how the estimated rate of occurrence (i.e. the intensity function) of heat stress events in the EU, Russia, Ukraine and India has increased in the last decades. On the other hand, it has remained almost constant in Canada and China (that has only recently experienced these large scale extremes) and it has decreased in Australia. In the U.S. the identified temporal pattern could be the net effect of large-scale irrigation and climate change^21^.

Concerning drought events, within each of the analysed region temporal inhibition is estimated (Fig. S3 in the Supplementary material). Moreover, there is a lower degree of connectivity with respect to heat stress events, with dependence between: Canada and the U.S., Canada and India, Australia and the EU (Fig. S3 in the Supplementary material).

**Figure 4:**
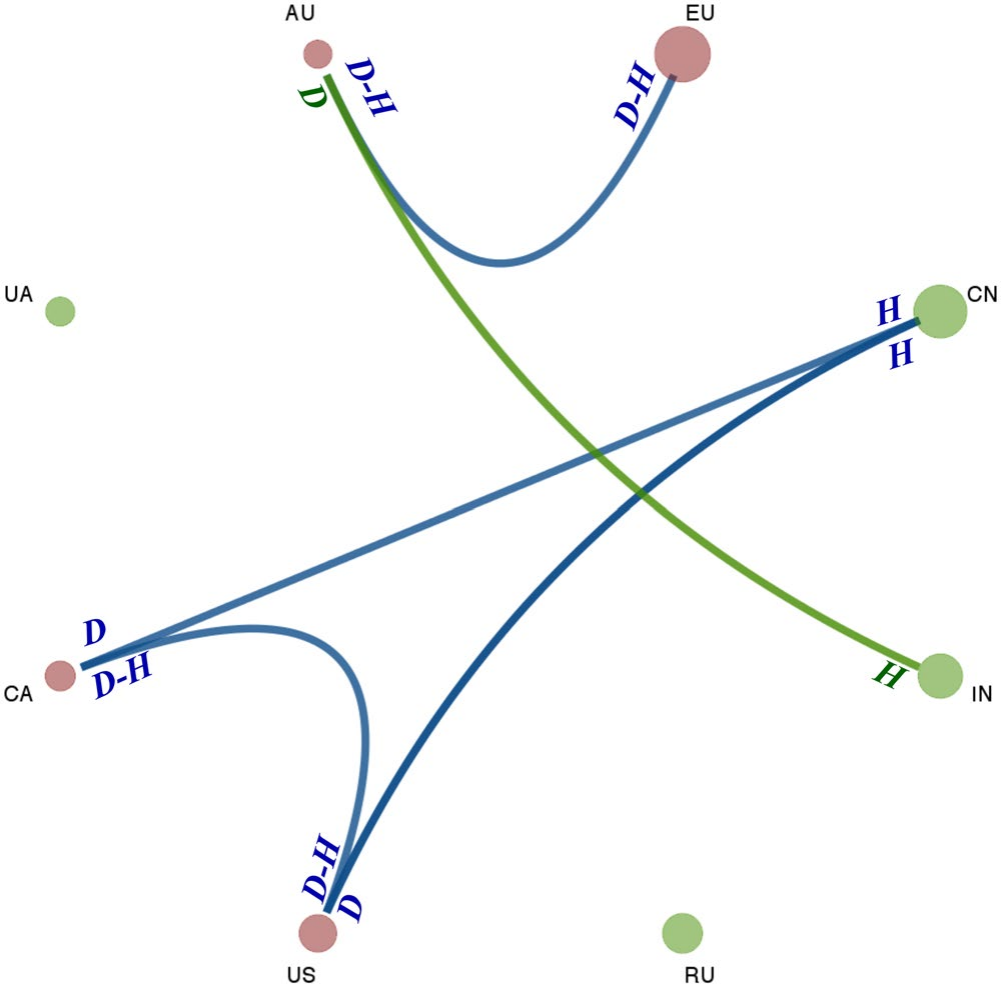
Summary plot of the analysis on concurrent drought (D) and heat stress (H) events. Disks represent the 8 key regions, with the size being proportional to the corresponding wheat production in 2016 (data from FAOSTAT). Green disks represent regions having no event (heat and drought) dependence within the region itself, while red ones represent regions with temporal clustering within the region itself. Blue links represent estimated clustering between regions, while green links represent estimated inhibition between regions. For instance, a blue link with D-H means that there is both drought-heat and heat-drought clustering, while D in one side of the link and H in the other one show estimated clustering/inhibition (according to the colour of the link) between drought events in one region and heat stress events in the other one.

The analysis of drought and heat stress interactions reveals how these events are closely interlinked in the EU, Australia, Canada and the U.S. (Fig. 4), all showing temporal dependence of these two types of events. While in the other regions (Ukraine, Russia, China, and India) our results suggest independence between heat stress and drought events (Fig. 4). These findings, despite not providing an exhaustive description of the causality of the events and their dynamics, are coherent with previous ones showing the important role of soil desiccation and soil-atmosphere feedback in triggering extreme heat events^22^; on the other side, they also show that drought and extreme heat are not necessarily linked. From an agronomic point of view, the identified temporal dependencies are of high interest (especially considering future climate projections^23,24^) as combined drought and heat stress have been recently shown to strongly affect photosynthetic carbon uptake and water use efficiency of wheat^25^. Figure 4 also shows interesting clustering-dependences emerging between drought and heat stress events in the EU wheat producing area and the Australian one, and between the U.S. and Canada. Clustering dependences are also identified for drought events occurring in the U.S. and Canada and large-scale heat stress events in China. Despite the spatial proximity, no clear significant dependence-structure has emerged between events in Russia and Ukraine, supporting random occurrence of drought events in one country and heat stress in the other one and vice versa. It is worth noting that the identified dependence of drought and heat stress events between Australia and the EU is coherent with the recent extremes of 2018, where extreme drought and heat stress were observed in both regions.

The identified dependencies in different regions of the world point to the need of integrating such information in global impact assessment of agricultural markets. As shown by Chatzopoulos *et al*.^26^, single isolated climate extremes in a key producing country can induce global price spikes and modify trade patterns with effects going beyond the year of occurrence. Thus, concurrent extremes could induce unpredicted cascades of shocks with serious and long-lasting socio-economic consequences (e.g. price volatility, spikes, and trade restrictions), even provoking instability and exacerbating hunger in countries depending on food imports^27,28^.

From a climate perspective, some of the emerging links are somewhat expected due to the spatial closeness of the regions, e.g, Russia-Ukraine, the U.S.-Canada. While understanding the nature of all the other ones (i.e. the large-scale climate processes behind them) is not trivial as it requires a comprehension of the global climate teleconnections (in terms of interactions and spatio-temporal scales) that goes beyond the current knowledge^29^. The connection between the North Pacific Oscillation (NPO) and El Niño Southern Oscillation (ENSO) modulated by the Arctic Oscillation (AO)^30^, the circumglobal and the Atlantic-Eurasian teleconnections^31^, the connection between the Walker circulation and the extratropical waves^32^ may all contribute to the emerging links between large-scale extremes. The dependence between large-scale drought and heat stress events in Australia and the EU might be induced, for instance, by the seasonal footprint mechanism linking NPO and ENSO and its modulation via the AO^30^. Indeed, with spring positive AO (favouring dry and warmer conditions especially in the southern part of Europe), positive NPO can trigger significant ENSO anomalies favouring dry conditions in Australia^29,33^. However, it must be noticed that a higher degree of complexity is realistically behind these links, likely to be characterised by nonlinearities. Concerning the case of EU-Australia link, for instance, the role of the Indian Ocean Dipole should also be taken into account^29,33–35^.

## Discussion

Taking into account the spatio-temporal dependence of climate extremes is essential for robust risk assessment. Concurrent extremes can have severe impacts in globally interconnected socio-economic sectors such as agriculture. The proposed methodological approach can identify such spatio-temporal structures without relying on strong (often unrealistic) assumptions. It could be used prior to other analyses and could guide the inference in (semi)parametric models such as the ones based on copulas. Despite its theoretical simplicity, the application of the method has higher complexity, as it requires random perturbation and double testing to take into account the lack of symmetry in the *J*-function. The dependence on the spatial threshold (used to define the large-scale events) is in fact of added value since it enables one to investigate the strength of the large-scale processes, and how their associated teleconnections may trigger extremes and induce dependencies. However, automatic techniques to identifying *J*-function departure from the Poisson process benchmark should be looked into, in order to reduce the degree of subjectiveness.

The application to extremes (heat stress and drought events) that have occurred in the key wheat producing regions of the world shows the importance of identifying concurrent climate extremes. Risk assessment and worst-scenario analyses^10^ aiming at quantifying the socio-economic impacts (as well as the system resilience) of shocks induced by climate extremes should not prescind from a robust dependence analysis. As shown by our findings, not considering the dependence effects can lead to serious underestimation of the impacts on the global agricultural system and to overconfidence in its resilience.

Finally, this study highlights the need for achieving a more coherent understanding of the global climate teleconnections, their interactions at different spatio-temporal scales and the induced extremes.

## Methods

The extremes have been derived using AgMERRA-temperature data^36^ and the Standardised Precipitation Evapotranspiration Index (SPEI) dataset^20^, while wheat data have been retrieved from MIRCA2000^37^. Following Zampieri *et al*.^3^, heat stress events at the grid scale have been identified by using the Heat Magnitude Day and then by applying thresholding at the 95^*th*^ percentile (i.e. defining *z*_*c*_). The Heat Magnitude Day (HMD) cumulates the effects of all heat waves occurring in the critical phenological phase (here, 3 months before harvesting), with each of them being characterised by

$${Z}_{heat,r}(x,t)=\sum _{i\in {D}_{t}(x)|{T}_{max,i}(x)\ge {T}_{max}^{90}(x)}\frac{{T}_{max,i}(x)-{T}_{max}^{25}(x)}{{T}_{max}^{75}(x)-{T}_{max}^{25}(x)}.$$

Here *T*_*max*_(*x*) represents the daily maximum temperature at the location *x*, *t* represents the year, *D*_*t*_(*x*) all days of the 3-month period before harvesting at the location *x* in the year *t* and the superscripted versions denote the corresponding percentiles. Recalling that we threshold HMD to obtain a set of spatial locations, to identify large-scale events a spatial threshold of 20% of wheat areas has been used for each of the 8 regions, i.e. *s*_*c*_ has been set at 20%; the measure *m* used here is the actual size. If the threshold has been crossed, we say that an event has occurred in year *t*. This implies that we focus on years having had heat extremes affecting a large part of the wheat producing area. This choice filters out smaller scale events that should not trigger any significant impact on the agricultural markets. Furthermore, such smaller scale events are likely to be independent from each other and induced by local climate conditions. Regarding SPEI, a similar procedure has been applied to identify large-scale drought events by thresholding at −1 (i.e. defining *z*_*c*_) at grid level and then by 20% of the wheat producing area of a region (i.e. defining *s*_*c*_).

Turning to the statistical methodology, denote by $|Y\cap A\times B\times C|$ the random number (cardinality) of events with marks belonging to $B\times C\subseteq {\mathscr{W}}\times {\mathscr{C}}$, which occur in the time set $A\subseteq  {\mathcal R}$. The intensity function of *Y* is defined as the function $\rho (\,\cdot \,)\ge 0$ satisfying

$$ {\mathcal E} [|Y\cap A\times B\times C|]=\sum _{r\in B}\sum _{c\in C}{\int }_{A}\rho (t,r,c)dt$$

for any $A\times B\times C\subseteq  {\mathcal R} \times {\mathscr{W}}\times {\mathscr{C}}$. Here, ${\mathcal E} [\,\cdot \,]$ denotes expectation and we note that *ρ*(*t*, *r*, *c*) may be interpreted as the density function of the probability that *Y* has a marked point in the infinitesimal region $dt\times \{r\}\times \{c\}\subseteq  {\mathcal R} \times {\mathscr{W}}\times {\mathscr{C}}$.

We proceed with the assumption of inhomogeneity, i.e. that the intensity function is non-constant, since the intensity of extreme events can vary over time. We further assume that the dependence between a collection of points of *Y* is governed by the respective inter-point distances as well as the underlying intensity.

The intensity function of the marked temporal point process *Y* can be estimated by applying a kernel-based estimator:

$${\hat{\rho }}_{r,c}(t;h)={h}^{-1}\sum _{y\in {Y}_{r,c}}{\mathscr{K}}(\frac{t-y}{h}),\,t\in [0,T],h > 0,$$

where ${\mathscr{K}}$ is a kernel function (a symmetric probability density), [0, *T*] is the observation interval and *h* = *h*_*r,c*_ > 0 is the smoothing bandwidth, which we select by following Cronie and van Lieshout^38^. Note that each pair $(r,c)\in {\mathscr{W}}\times {\mathscr{C}}$ generates an individual unmarked point process *Y*_*r*,*c*_ and thereby an individual intensity estimator, so that $\hat{\rho }(t,r,c)={\hat{\rho }}_{r,c}(t;{h}_{r,c})$.

Given non-empty $B\subseteq {\mathscr{W}}$ and $C\subseteq {\mathscr{C}}$, consider

$${f}_{B,C}(d,s,Y;\rho (\,\cdot \,))=\prod _{(t,r,c)\in Y\cap [s-d,s+d]\times {B}_{l}\times {C}_{m}}(1-\frac{{\bar{\rho }}_{B,C}}{\rho (t,r,c)}),\,s\in  {\mathcal R} ,d\ge 0,$$

where ${\bar{\rho }}_{B,C}$ is a positive constant such that ${\bar{\rho }}_{B,C}\le \rho (t,r,c)$ for all $(t,r,c)\in  {\mathcal R} \times B\times C$. A non-parametric estimator of the marked inhomogeneous empty space function is given by^19^

$${\hat{F}}_{B,C}(d)=1-\frac{1}{|L\cap [d,T-d]|}\sum _{s\in L\cap [d,T-d]}{f}_{B,C}(d,s,Y;\hat{\rho }(\,\cdot \,)),\,d < T/2,$$

where *L* is a fine point grid on [0, *T*]; ${\hat{F}}_{B,C}(d)=1$ for *d* ≥ *T*/2. Similarly, a non-parametric estimator of the marked inhomogeneous nearest neighbour distance distribution function is given by^19^

$${\hat{G}}_{({B}_{1},{C}_{1})\to ({B}_{2},{C}_{2})}(d)=1-\frac{1}{(T-2d)|{B}_{2}||{C}_{2}|}\sum _{r\in {B}_{2}}\sum _{c\in {C}_{2}}\sum _{s\in {Y}_{r,c}}{f}_{{B}_{1},{C}_{1}}(d,s,Y\backslash \{(s,r,c)\};\hat{\rho }(\,\cdot \,))$$

for *d* < *T*/2; ${\hat{G}}_{({B}_{1},{C}_{1})\to ({B}_{2},{C}_{2})}(d)=1$ for *d* ≥ *T*/2. Hence, we estimate ${J}_{({B}_{1},{C}_{1})\to ({B}_{2},{C}_{2})}(d)$, 0 ≤ *d* < *T*/2, by means of^19^

$$\frac{1-{\hat{G}}_{({B}_{1},{C}_{1})\to ({B}_{2},{C}_{2})}(d)}{1-{\hat{F}}_{{B}_{2},{C}_{2}}(d)};$$

${\hat{J}}_{({B}_{1},{C}_{1})\to ({B}_{2},{C}_{2})}(d)=1$ for *d* ≥ *T*/2. Note how the above expressions simplify when the mark sets all have cardinality 1.

All data used in this study are freely available and can be retrieved from the websites reported in the associated references.

## Supplementary information

Supplementary Material

## References

[1] EM-DAT, The Emergency Events Database, *Université catholique de Louvain (UCL) - CRED, D. Guha-Sapir* - www.emdat.be, Brussels, Belgium (2018).

[2] Zampieri M, Ceglar A, Dentener F, Toreti A (2018). Understanding and reproducing regional diversity of climate impacts on wheat yields: current approaches, challenges and data driven limitations. Environ. Res. Lett..

[3] Zampieri M, Ceglar A, Dentener F, Toreti A (2017). Wheat yield loss attributable to heat waves, drought and water excess at the global, national and subnational scales. Environ. Res. Lett..

[4] Rezaei EE, Webber H, Gaiser T, Naab J, Frank E (2015). Heat stress in cereals: mechanisms and modelling. Eur. J. Agron..

[5] Siebert S, Ewert F (2014). Future crop production threatened by extreme heat. Environ. Res. Lett..

[6] Lobell DB, Tebaldi C (2014). Getting caught with our plants down: the risks of a global crop yield slowdown from climate trends in the next two decades. Environ. Res. Lett..

[7] Fahad S (2017). Crop Production under Drought and Heat Stress: Plant Responses and Management Options. Front. Plant. Sci..

[8] Rezaei EE, Webber H, Gaiser T, Naab J, Ewert F (2015). Heat stress in cereals: Mechanisms and modelling, Europ. J. Agronomy.

[9] Lesk C, Rowhani P, Ramankutty N (2016). Influence of extreme weather disasters on global crop production. Nature.

[10] The Global Food Security programme, Extreme weather and resilience of the global food system (Final Project Report from the UK-US Taskforce on Extreme Weather and Global Food System Resilience, UK, 2015).

[11] Zampieri M (2016). Global assessment of heat wave magnitudes from 1901 to 2010 and implications for the river discharge of the Alps. Sci. Total Environ..

[12] Vicente-Serrano SM (2010). A multi-scalar drought index sensitive to global warming: the standardized precipitation evapotranspiration index. J. Climate.

[13] Toreti A, Giannakaki P, Martius O (2016). Precipitation extremes in the Mediterranean region and associated upper-level synoptic-scale flow structures. Clim. Dynam..

[14] Davison AC, Huser R (2015). Statistics of Extremes. Annu. Rev. Stat. Appl..

[15] Aghakouchak A, CHeng L, Mazdiyasni O, Farahmand A (2014). Global warming and changes in risk of concurrent climate extremes: insights from the 2014 California drought. Geophys. Res. Lett..

[16] Zscheischler J (2018). Future climate risk from compound events. Nat. Clim. Change.

[17] Daley, D. J. & Vere-Jones, D. An Introduction to the Theory of Point Processes: Volume I: Elementary Theory and Methods (2nd edn, Springer, New York, 2003).

[18] Daley, D. J. & Vere-Jones, D. *An Introduction to the Theory of Point Processes: Volume II: General Theory and Structure* (2nd edn, Springer, New York, 2008).

[19] Cronie O, van Lieshout MNM (2016). Summary statistics for inhomogeneous marked point processes. Ann. Inst. Stat. Math..

[20] Vicente-Serrano SM, Begueria S, Lopez-Moreno JI, Angulo-Martinez M, El Kenawy AM (2010). A new global 0.5° gridded dataset (1901–2006) of a multiscalar drought index: comparison with current drought index datasets based on the Palmer Drought Severity Index. J. Hydrometeorol..

[21] Bonfils C, Lobell D (2007). Empirical evidence for a recent slowdown in irrigation-induced cooling. P. Natl. Acad. Sci. USA.

[22] Miralles DG, Teuling AJ, van Heerwaarden CC, Vil’a-Guerau de Arellano J (2014). Mega-heatwave temperatures due to combined soil desiccation and atmospheric heat accumulation. Nat. Geosci..

[23] Russo S (2014). Magnitude of extreme heat waves in present climate and their projection in a warming world. J. Geophys. Res..

[24] Carrão H, Naumann G, Barbosa P (2018). Global projections of drought hazard in a warming climate: a prime for disaster risk management. Clim. Dyn..

[25] Urban O (2018). Combined effects of drought and high temperature on photosynthetic characteristics in four winter wheat genotypes. Field Crops Res.

[26] Chatzopoulos, T., Perez-Dominguez, I., Zampieri, M. & Toreti, A. Climate extremes and agricultural commodity markets: a global economic analysis of regionally simulated events, *Weather and Climate Extremes*, in press (2019).

[27] Bren d’Amour C, Wenz L, Kalkuhl M, Steckel JC, Creutzig F (2016). Teleconnected food supply shocks. Environ. Res. Lett..

[28] Puma MJ, Bose S, Chon SY, Cook BI (2015). Assessing the evolving fragility of the global food system. Environ. Res. Lett..

[29] Steptoe H, Jones SEO, Fox H (2018). Correlations between extreme atmospheric hazards and global teleconnections: implications for multihazard resilience. Rev. Geophys..

[30] Chen S, Chen W, Yu B, Graf H-F (2013). Modulation of the seasonal footprinting mechanism by the boreal spring Arctic Oscillation. Geophys. Res. Lett..

[31] Li J, Ruan C (2018). The North Atlantic-Eurasian teleconnection in summer and its effects on Eurasian climates. Env. Res. Lett..

[32] Liess S, Agrawal S, Chatterjee S, Kumar V (2017). A teleconnection between the West Siberian Plain and the ENSO region. J. Climate.

[33] Lim E-P, Hendon HH, Zhao M, Yin Y (2017). Inter-decadal variations in the linkage between ENSO, the IOD and south-eastern Australia springtime rainfall in the past 30 years. Clim. Dyn..

[34] Ummenhofer CC (2011). Indian and Pacific Ocean Influences on Southeast Australian Drought and Soil Moisture. J. Climate.

[35] Behera S, Ratnam JV, Masumoto Y, Yamagata T (2013). Origin of extreme summers in Europe: the Indo-Pacific connection. Clim. Dyn..

[36] Ruane AC, Goldberg R, Chryssanthacopoulos J (2015). AgMIP climate forcing datasets for agricultural modeling: merged products for gap-filling and historical climate series estimation. Agr. Forest Meteorol..

[37] Portmann FT, Siebert S S, Döll P (2010). MIRCA2000 Global monthly irrigated and rainfed crop areas around the year 2000: a new high-resolution data set for agricultural and hydrological modeling. Glob. Biogeochem. Cycles.

[38] Cronie O, van Lieshout MNM (2018). A non-model-based approach to bandwidth selection for kernel estimators of spatial intensity functions. Biometrika.

