# Supplementary material for: Concurrent climate extremes in the key wheat producing regions of the world
Source: Sci Rep. 2019 Apr 2;9:5493. doi: 10.1038/s41598-019-41932-5 (PMC6445097; doi:10.1038/s41598-019-41932-5)
Supplement: Supplementary file 1 — Supplementary Material [file 41598_2019_41932_MOESM1_ESM.pdf]

Supplementary Material
Concurrent climate extremes in the key wheat producing regions of the world

Andrea Toreti,[1*] Ottmar Cronie,[2] Matteo Zampieri[1]

[1]European Commission, Joint Research Centre (JRC), Ispra, Italy
[2]Department of Mathematics & Mathematical Statistics, Umeå University, Umeå, Sweden

[*]Andrea Toreti, E-mail: andrea.toreti@ec.europa.eu

Figures S1 and S2 show two examples on the use and interpretation of the J-function. In Figure S1, the estimated J functions point to inhibition between heat stress events occurring in the EU and India. The time scale is 0-3 years as shown by the lower panel of Figure S1. While, the estimated J-functions shown in Figure S2 support independence between heat stress events occurring in India and Russia. Figure S3 summaries the analysis of large scale drought events in the 8 key wheat producing regions of the world. The estimated intensities point to an increase in the occurrence of these extreme events in Australia especially in the last 10 years; while a decreasing tendency is estimated for the events in Canada and the U.S. (the latter one affected by higher uncertainty). Concerning all the other regions, a peak in the occurrence seems to have been reached in the 1990s, followed by either a decrease of a stabilisation (Fig. S3). Finally, the dependence analysis support a clustering behaviour between the EU and Australia, Canada and the U.S., and India and Canada (Fig. S3).

Region	*Acronym*	*Area (Mha)*	*Production (Mtonnes)*
European Union	EU	27	143
China	CN	24	132
India	IN	30	93
Russia	RU	27	73
USA	US	18	63
Canada	CA	9	30
Ukraine	UA	6	26
Australia	AU	11	22

Table S1. 2016 Wheat harvested area and production in the 8 key regions (data from FAOSTAT).

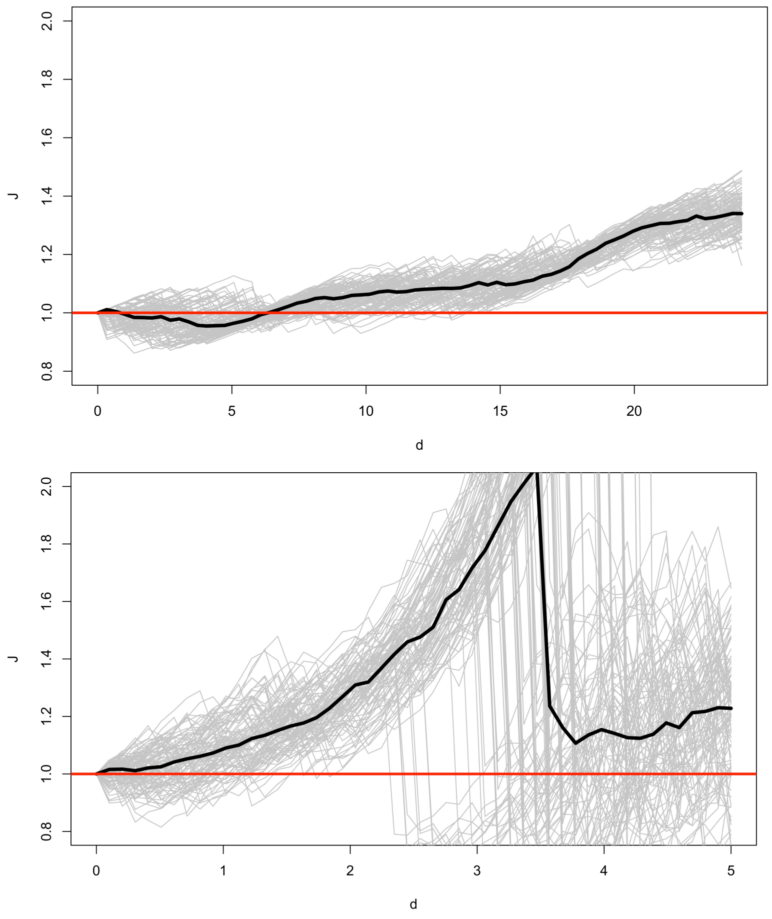

Figure S1. Estimated J-functions for heat stress events occurring in the EU and India (conditioning on the EU-events) showing inhibition. The upper panel shows the J-function obtained by perturbing at the monthly scale, the lower panel the one obtained with the perturbation at the annual scale.

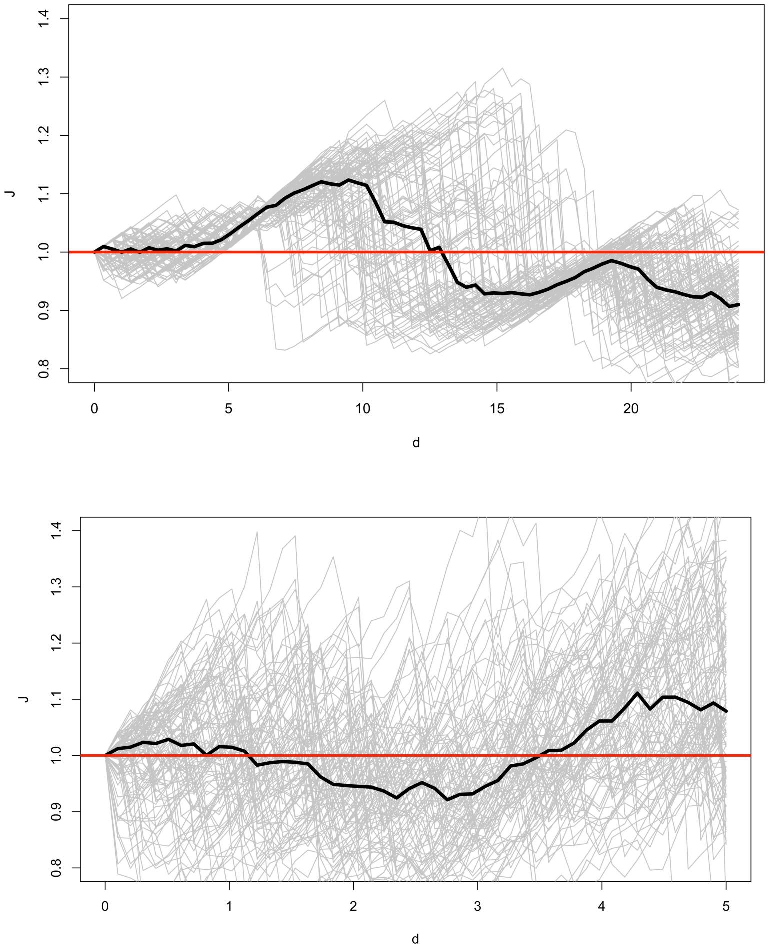

Figure S2. Estimated J-functions for heat stress events occurring in India and Russia (conditioning on India-events) showing no clear departure from Poisson process behaviour. The upper panel shows the J-function obtained by perturbing at the monthly scale, the lower panel the one obtained with the perturbation at the annual scale.

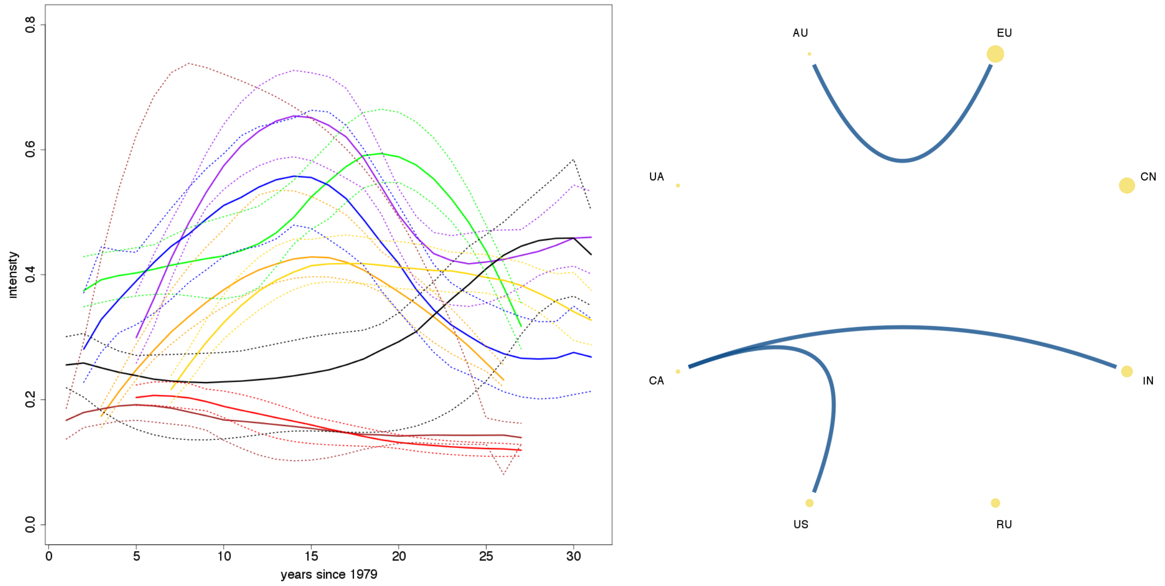

Figure S3. Left panel: estimated intensity functions (median in bold) and associated maximum and minimum of the ensemble (dotted lines) for drought events in the key wheat producing regions. Colours are associated with the 8 regions as in Fig.1. Right panel: summary plot of the analysis on drought events. Disks represent the 8 key regions with the size being proportional to the wheat production in 2016 (data from FAOSTAT). Yellow disks represent regions with temporal inhibition within the region itself. Blue links represent estimated clustering between regions.
